# Myofibrillar and Mitochondrial Protein Synthesis Rates Do Not Differ in Young Men Following the Ingestion of Carbohydrate with Milk Protein, Whey, or Micellar Casein after Concurrent Resistance- and Endurance-Type Exercise

**DOI:** 10.1093/jn/nxy244

**Published:** 2019-01-29

**Authors:** Tyler A Churchward-Venne, Philippe J M Pinckaers, Joey S J Smeets, Wouter M Peeters, Antoine H Zorenc, Henk Schierbeek, Ian Rollo, Lex B Verdijk, Luc J C van Loon

**Affiliations:** 1 NUTRIM School of Nutrition and Translational Research in Metabolism, Department of Human Biology, Maastricht University Medical Center+, Maastricht, Netherlands; 2 Department of Pediatrics, Academic Medical Center, Emma Children's Hospital, Amsterdam, Netherlands; 3 Gatorade Sports Science Institute, Leicester, United Kingdom

**Keywords:** muscle protein synthesis; young men; carbohydrate, dietary protein; milk; whey; micellar casein; concurrent exercise

## Abstract

**Background:**

Whey and micellar casein are high-quality dairy proteins that can stimulate postprandial muscle protein synthesis rates. How whey and casein compare with milk protein in their capacity to stimulate postprandial myofibrillar (MyoPS) and mitochondrial (MitoPS) protein synthesis rates during postexercise recovery is currently unknown.

**Objective:**

The objective of this study was to compare postprandial MyoPS and MitoPS rates after protein-carbohydrate co-ingestion with milk protein, whey, or micellar casein during recovery from a single bout of concurrent resistance- and endurance-type exercise in young healthy men.

**Methods:**

In a randomized, double-blind, parallel-group design, 48 healthy, young, recreationally active men (mean ± SEM age: 23 ± 0.3 y) received a primed continuous infusion of L-[ring-^13^C_6_]-phenylalanine and L-[ring-3,5-^2^H_2_]-tyrosine and ingested 45 g carbohydrate with 0 g protein (CHO), 20 g milk protein (MILK), 20 g whey protein (WHEY), or 20 g micellar casein protein (CASEIN) after a sequential bout of resistance- and endurance-type exercise (i.e., concurrent exercise). Blood and muscle biopsies were collected over 360 min during recovery from exercise to assess MyoPS and MitoPS rates and signaling through mammalian target of rapamycin complex 1 (mTORC1).

**Results:**

Despite temporal differences in postprandial plasma leucine concentrations between treatments (*P* < 0.001), MyoPS rates over 360 min of recovery did not differ between treatments (CHO: 0.049% ± 0.003%/h; MILK: 0.059% ± 0.003%/h; WHEY: 0.054% ± 0.002%/h; CASEIN: 0.059% ± 0.005%/h; *P* = 0.11). When MILK, WHEY, and CASEIN were pooled into a single group (PROTEIN), protein co-ingestion resulted in greater MyoPS rates compared with CHO (PROTEIN: 0.057% ± 0.002%/h; CHO: 0.049% ± 0.003%/h; *P* = 0.04). MitoPS rates and signaling through the mTORC1 pathway were similar between treatments.

**Conclusion:**

MyoPS and MitoPS rates do not differ after co-ingestion of either milk protein, whey protein, or micellar casein protein with carbohydrate during recovery from a single bout of concurrent resistance- and endurance-type exercise in recreationally active young men. Co-ingestion of protein with carbohydrate results in greater MyoPS, but not MitoPS rates, when compared with the ingestion of carbohydrate only during recovery from concurrent exercise. This trial was registered at Nederlands Trial Register: NTR5098.

## Introduction

Administration of mixed amino acids (1) or dietary protein (2) increases skeletal muscle protein synthesis (MPS) rates in a dose-dependent manner (3, 4) through activation of the mammalian target of rapamycin (mTOR) complex-1 (mTORC1) in vivo in humans (5). The effect of protein intake on MPS rates is further potentiated by resistance- (6, 7) or endurance-type (8, 9) exercise, resulting in MPS rates greater than those observed after protein ingestion or exercise only. Some (10–13), but not all (6, 14, 15), studies directly comparing postexercise MPS rates after ingestion of different types of protein have shown differences in the capacity of these proteins to stimulate MPS rates. The different anabolic properties of protein from different sources have been attributed to their protein digestion and/or absorption kinetics (16–18) and/or differences in their amino acid and leucine contents (11, 19). With regard to the latter, leucine content may be of particular relevance as leucine represents a key nutrient regulator of translation initiation (20) and can stimulate MPS in humans (21).

Bovine milk protein and its 2 major fractions, whey and micellar casein, are among the highest-quality sources of dietary protein based on current indexes of protein quality (22). Whey protein (∼20% of bovine milk protein) has a relatively high content of indispensable amino acids and is especially high in leucine (23), and has been characterized as a rapidly digested protein (16). Ingestion of whey protein results in a rapid, large, but transient increase in postprandial plasma indispensable amino acids and leucine availability (16). In contrast, micellar casein (∼80% of bovine milk protein) has a similar content of indispensable amino acids, but lower leucine content compared with whey, and has been characterized as a more slowly digested protein (16) as it coagulates and precipitates in the low pH environment of the stomach after ingestion (24). The resulting dairy curd is slowly released from the stomach resulting in a slower, moderate, but protracted hyperaminoacidemia (16). To date, studies comparing the capacity of different milk-derived proteins to stimulate postprandial MPS rates after exercise have focused on comparisons between whey and casein (6, 10, 11, 14, 15). Results from these studies either demonstrate a superior effect of whey (10, 11), or fail to detect any differences between proteins (6, 14, 15) in their capacity to increase postexercise MPS rates. To date, no studies have directly compared whey and micellar casein to milk protein in their capacity to increase postprandial MPS rates in humans after exercise. Milk protein, as a blend of whey and micellar casein, may offer an advantage because of the unique properties of both protein fractions.

The majority of studies examining nutrition and exercise interactions on MPS have focused on protein ingestion during recovery after resistance-type exercise (25). However, recreational athletes in a variety of sports and individuals who exercise for general health and fitness typically engage in exercise training that incorporates both resistance- and endurance-type exercise (i.e., concurrent exercise). As resistance- and endurance-type exercise exert differential effects on rates of myofibrillar protein synthesis (MyoPS) (26) and mitochondrial protein synthesis (MitoPS) (26), there is a need to better understand the interaction between protein ingestion and concurrent resistance- and endurance-type exercise on postexercise MyoPS and MitoPS rates. To date, only a few studies have investigated the impact of protein ingestion on postprandial MitoPS rates after exercise compared with exercise or protein ingestion only (9, 27–29); however, no studies have evaluated the potential impact of different types of protein on the synthetic rate of this muscle protein subfraction after exercise. The present study examined the effects of co-ingesting 20 g protein from milk, whey, or micellar casein with 45 g carbohydrate on postprandial MyoPS and MitoPS rates during recovery from a single bout of concurrent resistance- and endurance-type exercise in young, healthy, recreationally active men. We hypothesized that co-ingestion of protein with carbohydrate, regardless of protein source, would result in greater postexercise MyoPS and MitoPS rates than would ingestion of carbohydrate only, and that milk and whey would stimulate the greatest postexercise MyoPS and MitoPS rates.

## Methods

### Participants

Forty-eight healthy recreationally active young men (age 23 ± 0.3 y; height 1.80 ± 0.01 m; weight 74.2 ± 1.1 kg; values are mean ± SEM) volunteered to participate in this parallel group, double-blind, randomized controlled trial. “Recreationally active” was defined as engaging in sports or structured exercise 1–3 d/wk. The trial was registered at the Nederlands Trial Register (NTR5098), and was conducted between March 2015 and May 2016 at Maastricht University in Maastricht, Netherlands. Participants’ characteristics are presented in **Supplemental Table 1**. All participants were informed of the purpose of the study, the experimental procedures, and possible risks before providing informed written consent to participate. The procedures followed were in accordance with the ethical standards of the medical ethics committee of Maastricht University Medical Center+ on human experimentation and in accordance with the Helsinki Declaration of 1975 as revised in October 2013. The study was independently monitored by the Clinical Trial Centre Maastricht (CTCM).

### Preliminary testing

Participants aged 20–30 y inclusive, with a BMI >19.0 and <25.0 (in kg/m^2^) underwent an initial screening session to assess height, weight, blood pressure, and body composition (by dual-energy X-ray absorptiometry; Discovery A, Hologic). Participants were deemed healthy based on their responses to a medical questionnaire and screening results. After the assessment of baseline anthropometrics, participants were familiarized with the exercise testing protocol and the exercise equipment. All exercise testing during the preliminary testing visit was supervised by >1 of the study investigators. Participants underwent estimates of 1 repetition maximum (1-RM) strength on the supine leg press (Technogym BV) and seated leg extension (Technogym BV) exercise through the use of the multiple repetition testing procedure (30). Before testing each exercise, participants performed 10 submaximal repetitions to become familiarized with the equipment and to have exercise technique assessed and adjusted by 1 of the study investigators. Working sets were then performed with progressively increased loads until failure to perform a valid estimation within 3–6 repetitions of the set. A repetition was considered valid if the subject was able to complete it in a controlled manner as determined by a study investigator. A 2-min interset rest period was allowed between successive sets. After estimates of 1-RM on the leg press and leg extension exercise, peak power output was determined during an incremental test to volitional fatigue on a cycle ergometer (Lode BV). Participants began cycling at a workload equivalent to 2 W/kg body weight for 150 s, after which the workload was increased by 25 W every 150 s until volitional fatigue was reached, defined as the inability to maintain a cadence >60 revolutions/min. All equipment settings were noted and replicated during the experimental test day. The pretesting and experimental trials were separated by ≥5 d.

### Study design

Participants were randomly assigned to ingest a beverage (590 mL) containing 45 g of carbohydrate with 0 g protein (CHO), or 45 g of carbohydrate with 20 g milk protein (MILK), whey protein (WHEY), or micellar casein protein (CASEIN). The caloric content of the beverages was therefore not matched between treatments and was ∼80 kcal lower in the CHO treatment because of the absence of protein. The carbohydrate powder was supplied by PepsiCo Inc, and was composed of dextrose and maltodextrin. Milk protein concentrate (Refit MPC 80), whey protein concentrate (Nutri Whey 800F), and micellar casein protein isolate (Refit MCI 88) were obtained from FrieslandCampina DMV B.V. Details of the amino acid, protein, and carbohydrate contents of the nutritional treatment are shown in **Supplemental Table 2**. Random assignment was performed with a computerized list randomizer (https://www.random.org/lists/), and participants were sequentially allocated to a treatment according to the random assignment list.

### Diet and physical activity

All participants were instructed to refrain from strenuous physical activities and alcohol consumption for 3 d before the experimental trial. In addition, all participants were instructed to complete food intake and physical activity questionnaires for 2 d before the experimental trial. On the evening before the experimental trial, all participants were provided with a prepackaged standardized meal containing 55% energy as carbohydrate, 30% energy as fat, and 15% energy as protein, and instructed to consume it no later than 2000, after which they remained fasted.

### Experimental protocol

**FIGURE 1:**
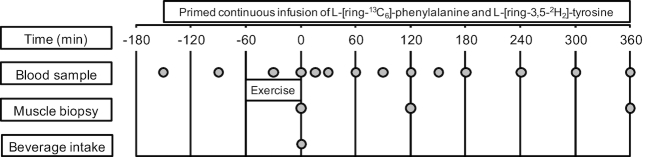
Schematic representation of the experimental design.

At ∼0745, participants arrived at the laboratory in the overnight postabsorptive state. A catheter was inserted into an antecubital vein for stable isotope amino acid infusion, while a second catheter was subsequently inserted into a dorsal hand vein on the contralateral arm for arterialized blood sampling. To obtain arterialized blood samples, the hand was placed in a hot box (60°C) for 10 min before blood sample collection (31). After taking a baseline blood sample (*t* = −150 min), the plasma phenylalanine pool was primed with a single dose of L-[ring-^13^C_6_]-phenylalanine (2.25 μmol/kg) and L-[ring-3,5-^2^H_2_]-tyrosine (0.867 μmol/kg), and a continuous intravenous infusion of L-[ring-^13^C_6_]-phenylalanine (0.05 μmol · kg^−1^ · min^−1^) and L-[ring-3,5-^2^H_2_]-tyrosine (0.019 μmol · kg^−1^ · min^−1^) was initiated (*t* = −150 min) with the use of a calibrated IVAC 598 pump. After resting in a supine position for 60 min, a second arterialized blood sample was drawn (*t* = −90 min). After resting for another 30 min, participants initiated (*t* = −60 min) the concurrent exercise intervention (described subsequently). A third blood sample was drawn (*t* = −30 min) during the transition from resistance- to endurance-type exercise. Immediately after the exercise intervention (*t* = 0 min), an arterialized blood sample was drawn and a muscle biopsy sample was collected from the vastus lateralis of a randomly selected leg. Subsequently, participants received a 590-mL beverage corresponding to their randomized treatment allocation [i.e., CHO (*n* = 12), MILK (*n* = 12), WHEY (*n* = 12), or CASEIN (*n* = 12)]. The beverages containing protein were enriched to 4% L-[ring-^13^C_6_]-phenylalanine to minimize dilution of the steady state plasma L-[ring-^13^C_6_]-phenylalanine precursor pool implemented by the constant infusion. Arterialized blood samples were then collected at *t* = 15, 30, 60, 90, 120, 150, 180, 240, 300, and 360 min in the postprandial period. A second and third muscle biopsy sample were collected at *t* = 120 and *t* = 360 min to determine postprandial MyoPS and MitoPS rates from *t* = 0–120, 120–360, and 0–360 min. Blood samples were collected into EDTA-containing tubes and centrifuged at 1000 ×*g* for 15 min at 4°C. Aliquots of plasma were frozen in liquid nitrogen and stored at −80°C. Biopsy samples were collected with the use of a 5-mm Bergström needle custom-adapted for manual suction. Samples were obtained from separate incisions from the middle region of the vastus lateralis, ∼15 cm above the patella and ∼3 cm below entry through the fascia, under 1% xylocaine local anesthesia with adrenaline (1:100,000). Muscle samples were freed from any visible nonmuscle material, immediately frozen in liquid nitrogen, and stored at −80°C until further processing. When the experimental protocol was complete, cannulae were removed and subjects were fed and assessed for ∼30 min before leaving the laboratory. For a schematic representation of the infusion protocol, see **Figure 1**.

### Concurrent exercise protocol

#### Resistance-type exercise

Participants began with a standardized warm-up on the supine leg press (1 × 10 repetitions at ∼50% estimated 1-RM), followed by 4 sets of 8 repetitions at ∼80% of their previously estimated 1-RM. Participants then carried out the same exercise protocol (i.e., same number of sets and repetitions at percentage of estimated 1-RM) on the seated leg-extension machine. Each set was separated by 2 min of passive recovery during which the subject remained seated. Range of motion was set from ∼70° to 155° for the leg press and from ∼75° to 165° for the leg extension. Strong verbal encouragement was provided by 1 of the study investigators during each set.

#### Endurance-type exercise

After resistance-type exercise, participants performed 30 min of continuous cycling at ∼60% of their previously determined maximal workload. Participants were allowed ad libitum access to water during cycling. Visual feedback for pedal frequency (rotations per minute) and elapsed time were provided to participants and strong verbal encouragement was provided by 1 of the study investigators.

### Plasma and muscle tissue analyses

#### Plasma analysis

Details of analysis relating to the determination of plasma glucose, insulin, and amino acid concentrations as well as plasma L-[ring-^13^C_6_]-phenylalanine, L-[ring-^13^C_6_]-tyrosine, and L-[ring-3,5-^2^H_2_]-tyrosine enrichments are presented in **Supplemental Methods**.

#### Muscle analysis

A piece of wet muscle (∼100 mg) was homogenized on ice with the use of a Teflon pestle in ice-cold homogenization buffer (10 μL/mg; 1 M sucrose, 1 M Tris/HCl, 1 M KCl, 1 M EDTA) containing protease/phosphatase inhibitor cocktail tablets (Complete Protease Inhibitor Mini-Tabs; and PhosSTOP, Roche Applied Science). After ∼5*–*10 min of hand homogenization, the homogenate was centrifuged at 700 × *g* for 15 min at 4°C to pellet a myofibrillar protein enriched fraction. The supernatant was transferred to another tube and centrifuged at 12,000 ×*g* for 20 min at 4°C to pellet a mitochondrial protein enriched fraction. The resulting supernatant was used for Western Blot analysis. Additional details regarding the preparation and analysis of skeletal muscle samples for measurement of myofibrillar and mitochondrial protein-bound phenylalanine enrichment, and intramuscular signaling via Western Blot are presented in **Supplemental Methods**.

### Calculations

The fractional synthetic rate (FSR) (%/h) of myofibrillar and mitochondrial protein enriched fractions was calculated by the standard precursor-product equation

(1) \begin{equation*}
{\rm{FSR}} = \left[ {\left( {{E_{2{{b}}}} - {E_{1{\mathop{b}\nolimits} }}} \right)/\left( {{E_{{\rm{precursor}}}} \times t} \right)} \right] \times 100
\end{equation*}

where *E_b_* is the increment in myofibrillar or mitochondrial protein-bound L-[ring-^13^C_6_]-phenylalanine enrichment (mole percentage of excess) between 2 muscle biopsy samples, *E*_precursor_ is the weighted mean plasma L-[ring-^13^C_6_]-phenylalanine enrichment (mole % excess) during the tracer incorporation period, and *t* is the tracer incorporation time in h. Weighted mean plasma enrichments were calculated by taking the measured enrichment between consecutive time points and correcting for the time between these sampling time points. For calculation of postprandial FSR, biopsy samples at *t* = 0, 120, and 360 min were used.

### Statistical analysis

Subjects’ characteristics, 1-RM strength, and maximal workload, were analyzed by a 1-factor (treatment) ANOVA. Blood glucose and plasma insulin were analyzed by a 2-factor (treatment × time) repeated-measures ANOVA. Plasma leucine, phenylalanine, and tyrosine concentrations were analyzed by a 2-factor (treatment × time) repeated-measures ANOVA. Leucine, phenylalanine, and tyrosine AUC were analyzed by a 1-factor (treatment) ANOVA. Plasma enrichments were analyzed by a 2-factor (treatment × time) repeated-measures ANOVA. Myofibrillar and mitochondrial FSR during early and late recovery (i.e., 0–120 and 120–360 min) and protein phosphorylation status (i.e., 0, 120, and 360 min) were analyzed by a 2-factor (treatment × time) repeated-measures ANOVA. The aggregate myofibrillar and mitochondrial FSR (i.e., 0–360 min) was analyzed by a 1-factor (treatment) ANOVA and independent samples *t* test. A power calculation was performed with differences in postprandial myofibrillar protein FSR as the primary outcome measure with the use of a standard deviation of 0.0065%/h in all treatments, and a difference in FSR of 0.008%/h between treatments (or ∼20% when expressed as relative difference between treatments). With a power of 80% and a significance level of 0.05, the final number of participants to be included was calculated as *n* = 12/group. Tukey's post hoc analysis was performed whenever a significant F ratio was found to isolate specific differences. Statistical analyses were performed with a software package (IBM SPSS Statistics for Windows, version 21.0, IBM Corp.). Means were considered to be significantly different for *P* values <0.05.

## Results

### Plasma analyses

**FIGURE 2:**
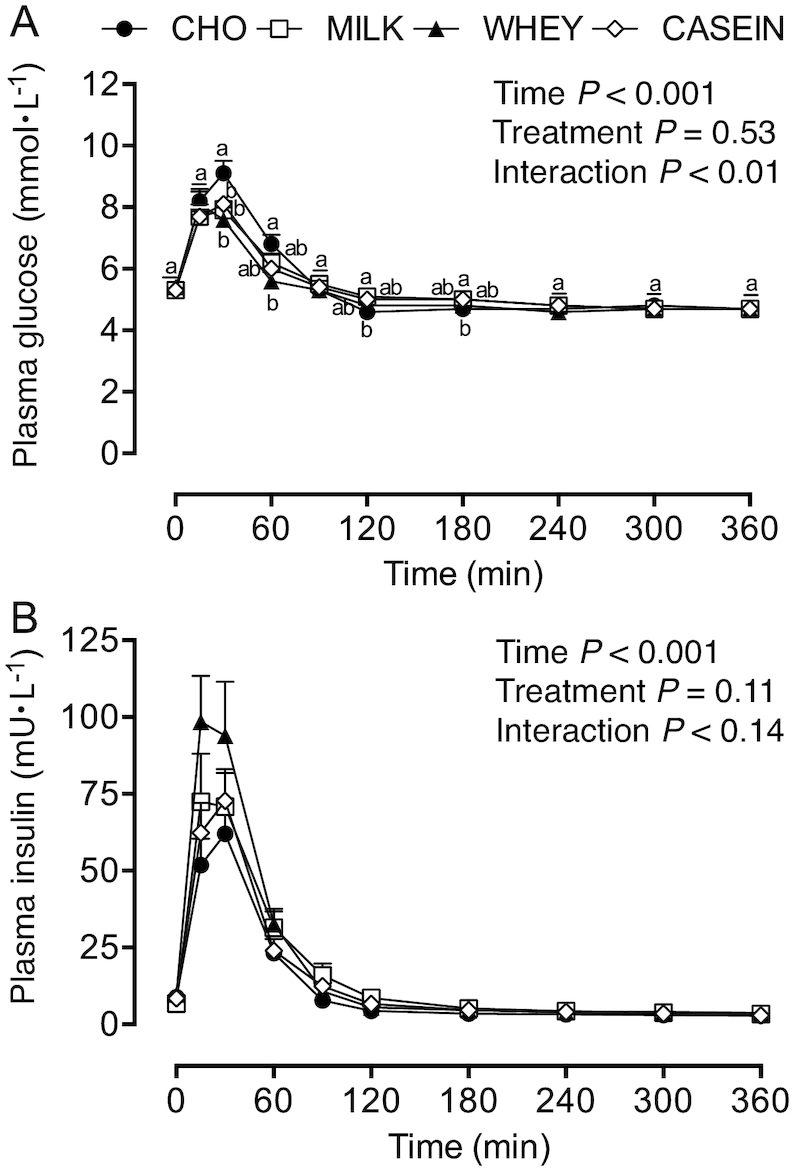
Plasma glucose (A) and insulin (B) concentrations during postabsorptive conditions (*t* = 0 min), and during postprandial conditions (*t* = 15–360 min) after beverage intake during recovery from a single bout of concurrent exercise in young men. Data for glucose and insulin were analyzed by a 2-factor repeated measures ANOVA. Values are means ± SEMs, *n* = 12. Labeled means within a time without a common letter differ, *P* < 0.05. CASEIN, 45 g carbohydrate co-ingested with 20 g micellar casein protein; CHO, 45 g carbohydrate with 0 g protein; MILK, 45 g carbohydrate co-ingested with 20 g milk protein; WHEY, 45 g carbohydrate co-ingested with 20 g whey protein.

Plasma glucose concentrations (**Figure 2**A) were transiently increased after each treatment (*P*-interaction < 0.01), with CHO resulting in higher concentrations than MILK, WHEY, and CASEIN at *t* = 30 min, and higher concentrations than WHEY at *t* = 60 min. At *t* = 120 and *t* = 180 min, plasma glucose concentrations were reduced compared with *t* = 0 min and were different between CHO and MILK. Plasma insulin concentrations (Figure 2B) were transiently increased during the postprandial period from *t* = 15–90 min (*P*< 0.001).

**FIGURE 3:**
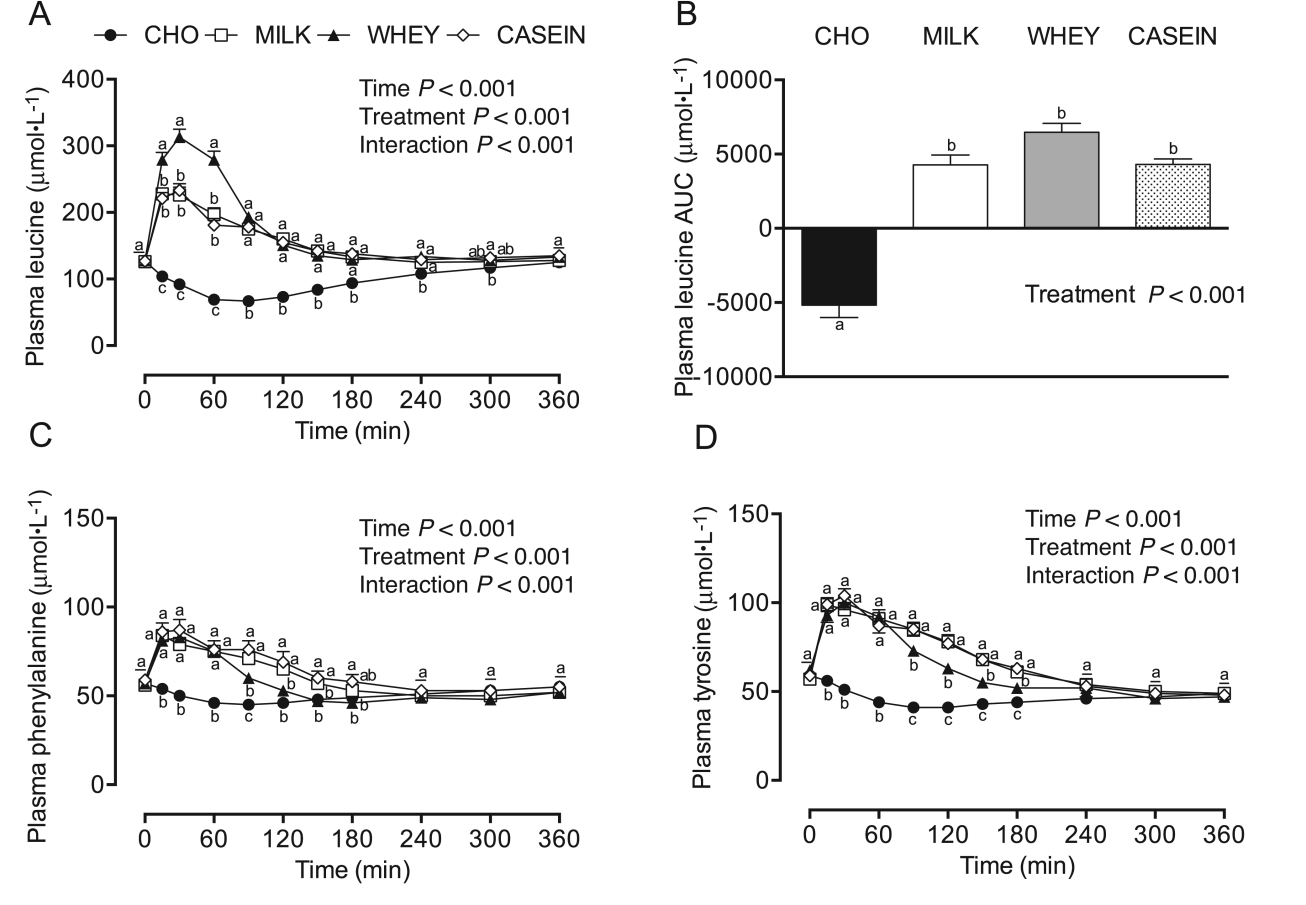
Plasma leucine (A), leucine AUC (B), phenylalanine (C), and tyrosine (D) concentrations during postabsorptive conditions (*t* = 0 min), and during postprandial conditions (*t* = 15–360 min) after beverage intake during recovery from a single bout of concurrent exercise in young men. Data for leucine, phenylalanine, and tyrosine were analyzed by a 2-factor repeated measures ANOVA. Data for leucine AUC were analyzed by a 1-factor ANOVA. Values are means ± SEMs, *n* = 12. Labeled means within a time without a common letter differ, *P* < 0.05. CASEIN, 45 g carbohydrate co-ingested with 20 g micellar casein protein; CHO, 45 g carbohydrate with 0 g protein; MILK, 45 g carbohydrate co-ingested with 20 g milk protein; WHEY, 45 g carbohydrate co-ingested with 20 g whey protein.

Plasma leucine concentrations (**Figure 3**A) were transiently increased after protein-carbohydrate co-ingestion, with WHEY resulting in higher leucine concentrations from *t* = 15–60 min compared with MILK and CASEIN. Plasma leucine concentrations in the MILK, WHEY, and CASEIN groups were increased when compared with the CHO group from *t* = 15–240 min, and remained increased in CASEIN compared with the CHO at *t* = 300 min during the postprandial period (*P*-interaction < 0.001). Leucine AUC (Figure 3B) was greater in MILK, WHEY, and CASEIN compared with CHO (*P* < 0.001), with WHEY showing a statistical trend for a greater leucine AUC compared with MILK (*P* = 0.09) and CASEIN (*P* = 0.09). Plasma phenylalanine concentrations (Figure 3C) were transiently increased after protein-carbohydrate co-ingestion, with MILK and CASEIN resulting in higher phenylalanine concentrations from *t* = 90–180 min compared with WHEY. Plasma phenylalanine concentrations were elevated in MILK, WHEY, and CASEIN when compared with CHO from *t* = 15–90 min, and remained elevated until *t* = 150 min for MILK and *t* = 180 min for CASEIN compared with CHO (*P*-interaction < 0.001). Plasma phenylalanine AUC (data not shown) were greater in MILK, WHEY, and CASEIN compared with CHO, and greater in MILK and CASEIN compared with WHEY (*P* < 0.001). Plasma tyrosine concentrations (Figure 3D) were transiently increased after protein-carbohydrate co-ingestion from *t* = 15–180 min compared with CHO (*P*-interaction < 0.001). Plasma tyrosine concentrations were increased in MILK and CASEIN when compared with WHEY from *t* = 90–180 min. Plasma tyrosine AUC (data not shown) were greater in MILK, WHEY, and CASEIN compared with CHO, and greater in MILK and CASEIN compared with WHEY (*P* < 0.001).

### Stable isotope tracer analyses

**FIGURE 4:**
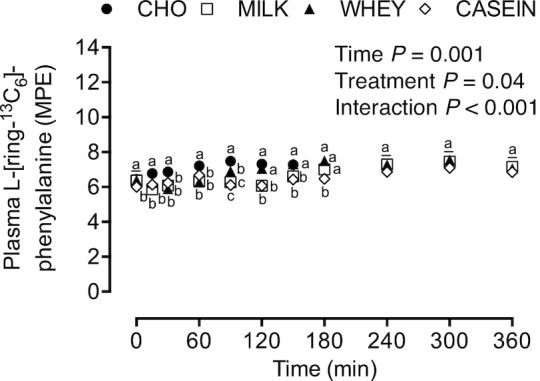
Plasma L-[ring-^13^C_6_]-phenylalanine enrichments during postabsorptive conditions (*t* = 0 min), and during postprandial conditions (*t* = 15–360 min) after beverage intake during recovery from a single bout of concurrent exercise in young men. Data were analyzed by a 2-factor repeated measures ANOVA. Values are means ± SEMs, *n* = 12. Labeled means within a time without a common letter differ, *P* < 0.05. CASEIN, 45 g carbohydrate co-ingested with 20 g micellar casein protein; CHO, 45 g carbohydrate with 0 g protein; MILK, 45 g carbohydrate co-ingested with 20 g milk protein; MPE, mole percentage excess; WHEY, 45 g carbohydrate co-ingested with 20 g whey protein.

Plasma L-[ring-^13^C_6_]-phenylalanine enrichments (**Figure 4**) were higher in CHO compared with MILK, WHEY, and CASEIN from *t* = 15–90 min, and remained higher when compared with MILK from *t* = 120–150 min and compared with CASEIN from *t* = 120–180 min. Plasma L-[ring-^13^C_6_]-phenylalanine enrichments were higher in WHEY when compared with MILK from *t* = 90–150 min and compared with CASEIN from *t* = 90–180 min (*P*-interaction < 0.001).

**FIGURE 5:**
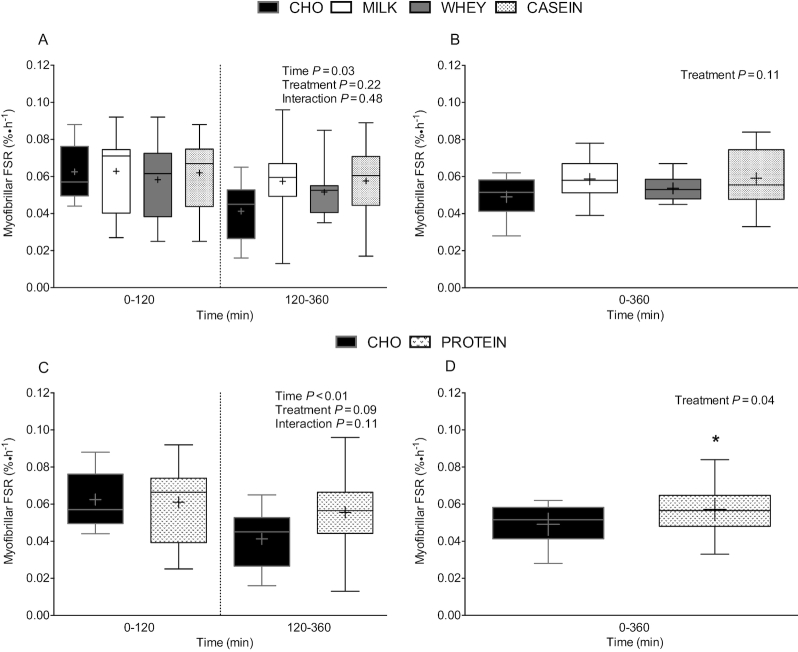
Myofibrillar protein FSR over 0–120 and 120–360 min (A and C), and over 0–360 min (B and D) after beverage intake during recovery from a single bout of concurrent exercise in young men. Time-course (Panel A and C) data were analyzed by a 2-factor repeated measures ANOVA. Aggregate (Panel B and D) data were analyzed by a 1-factor ANOVA (B) and independent samples *t* test (D). Boxes represent 25th to 75th percentiles. Horizontal lines and crosses within boxes represent medians and means, respectively. Whiskers represent minimums and maximums, *n* = 12. *Different from CHO, *P* < 0.05. CASEIN, 45 g carbohydrate co-ingested with 20 g micellar casein protein; CHO, 45 g carbohydrate with 0 g protein; FSR, fractional synthetic rate; MILK, 45 g carbohydrate co-ingested with 20 g milk protein; PROTEIN, 45 g carbohydrate co-ingested with 20 g protein (data collapsed across MILK, WHEY, and CASEIN); WHEY, 45 g carbohydrate co-ingested with 20 g whey protein.

**FIGURE 6:**
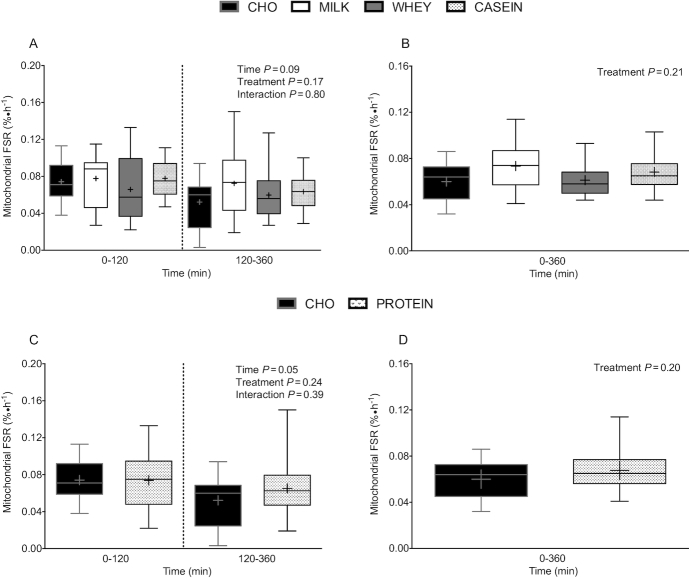
Mitochondrial protein FSR over 0–120 and 120–360 min (A and C), and over 0–360 min (B and D) after beverage intake during recovery from a single bout of concurrent exercise in young men. Time-course (panels A and C) data were analyzed by a 2-factor repeated measures ANOVA. Aggregate (panels B and D) data were analyzed by a 1-factor ANOVA (B) and independent samples *t* test (D). Boxes represent 25th to 75th percentiles. Horizontal lines and crosses within boxes represent medians and means, respectively. Whiskers represent minimums and maximums, *n* = 12. CASEIN, 45 g carbohydrate co-ingested with 20 g micellar casein protein; CHO, 45 g carbohydrate with 0 g protein; FSR, fractional synthetic rate; MILK, 45 g carbohydrate co-ingested with 20 g milk protein; PROTEIN, 45 g carbohydrate co-ingested with 20 g protein (data collapsed across MILK, WHEY, and CASEIN); WHEY, 45 g carbohydrate co-ingested with 20 g whey protein.

Postprandial MyoPS rates (i.e., myofibrillar FSR), assessed during early (0–120 min) and late (120–360 min) postexercise recovery (**Figure 5**A), did not differ between treatments (*P* = 0.22). Postprandial MyoPS rates were higher during early (0–120 min) compared with late (120–360 min) postexercise recovery (*P* = 0.03). Aggregate (0–360 min) postprandial MyoPS rates (Figure 5B) did not differ among treatments (*P* = 0.11). Given the lack of treatment effect, we collapsed data from MILK, WHEY, and CASEIN into a single treatment group (PROTEIN), to compare protein co-ingestion with carbohydrate ingestion only (CHO). Postprandial MyoPS rates, assessed during early (0–120 min) and late (120–360 min) postexercise recovery (Figure 5C), did not differ between PROTEIN and CHO (*P* = 0.09). Early MyoPS rates were higher than late MyoPS rates (*P* < 0.01). There was no interaction (*P* = 0.11). Aggregate (0–360 min) MyoPS rates (Figure 5D) were significantly higher in PROTEIN compared with CHO (*P* = 0.04). Postprandial MitoPS rates (i.e., mitochondrial FSR), assessed during early (0–120 min) and late (120–360 min) postexercise recovery (**Figure 6**A), did not differ between treatments (*P* = 0.17) or across time (*P* = 0.09). Similarly, aggregate (0–360 min) postprandial MitoPS rates (Figure 6B) did not differ among treatments (*P* = 0.21). Given the lack of treatment effect, we again collapsed data from MILK, WHEY, and CASEIN into a single treatment group (PROTEIN), to compare protein co-ingestion with carbohydrate ingestion only (CHO). Postprandial MitoPS rates, assessed during early (0–120 min) and late (120–360 min) postexercise recovery (Figure 6C), did not differ between PROTEIN and CHO (*P* = 0.24). However, early MitoPS rates were higher than late MitoPS rates (*P* = 0.05). Aggregate (0–360 min) MitoPS rates (Figure 6D) did not differ between PROTEIN compared with CHO (*P* = 0.20).

### Muscle tissue signaling

**FIGURE 7:**
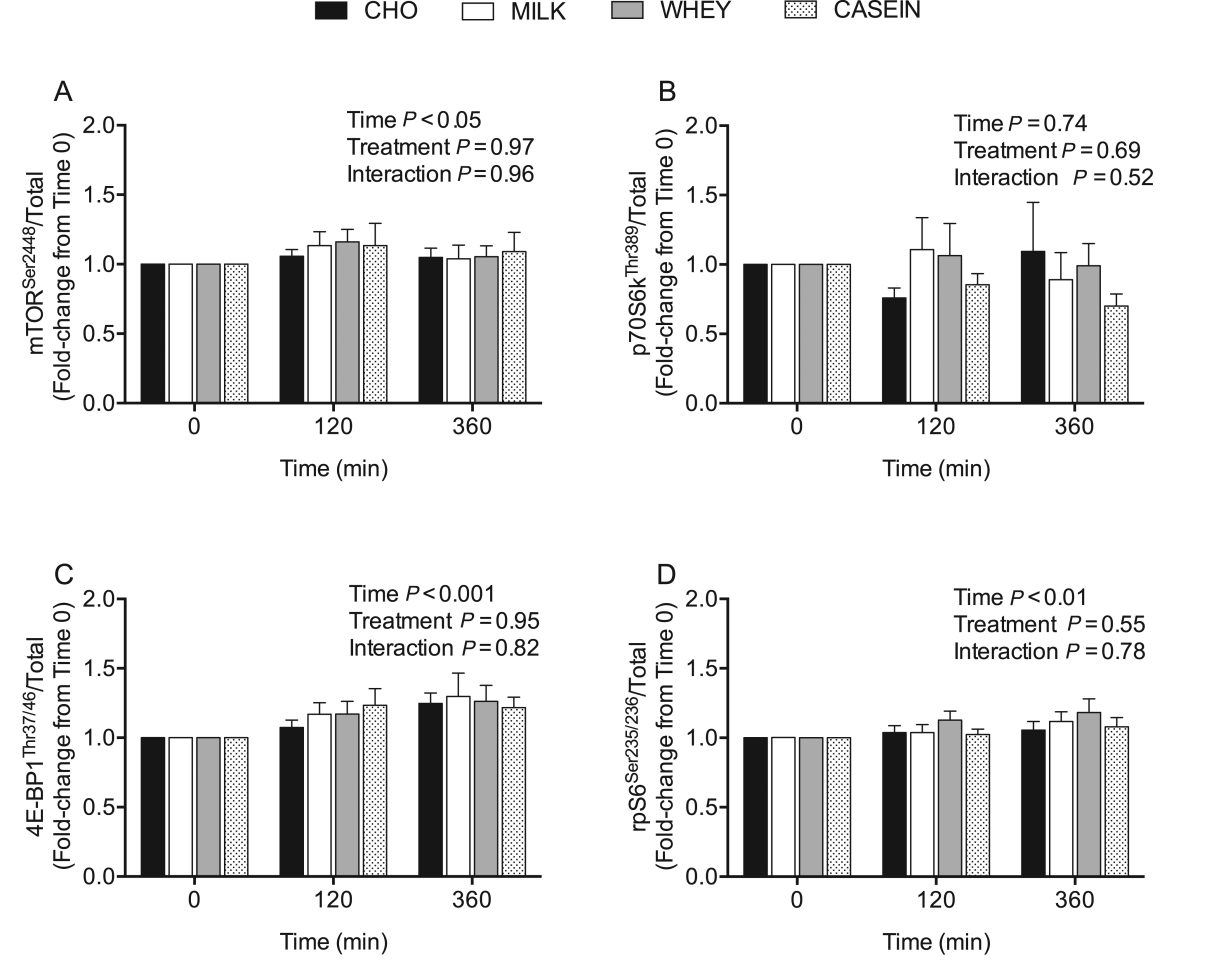
Phosphorylation of mTOR^Ser2448^ (A), p70S6k^Thr389^ (B), 4E-BP1^Thr37/46^ (C), and rpS6^Ser235/236^ (D) relative to the total abundance of their corresponding protein during postabsorptive conditions (*t* = 0 min), and during postprandial conditions (*t* = 120 and 360 min) after beverage intake during recovery from a single bout of concurrent exercise in young men. Data at *t* = 120 min and *t* = 360 min are expressed as fold-change from *t* = 0 min. Data were analyzed by a 2-factor repeated measures ANOVA. Values are means ± SEMs, *n* = 12. CASEIN, 45 g carbohydrate co-ingested with 20 g micellar casein protein; CHO, 45 g carbohydrate with 0 g protein; MILK, 45 g carbohydrate co-ingested with 20 g milk protein; mTOR, mammalian target of rapamycin; p70S6k, ribosomal protein S6 kinase; rpS6, ribosomal protein S6; WHEY, 45 g carbohydrate co-ingested with 20 g whey protein; 4E-BP1, eukaryotic initiation factor 4E binding protein.

**FIGURE 8:**
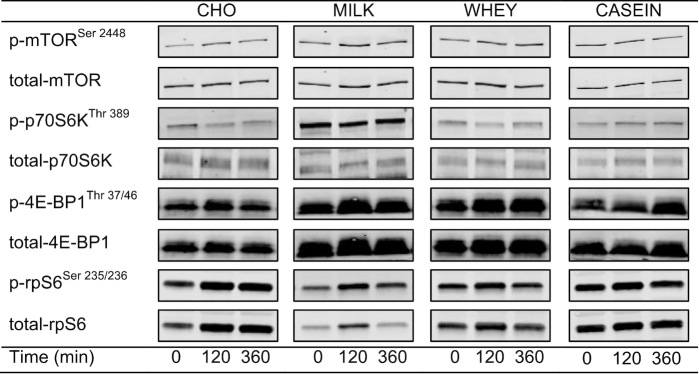
Representative Western Blot images for phosphorylated (p-) and total mTOR^Ser2448^, p70S6k^Thr389^, 4E-BP1^Thr37/46^, and rpS6^Ser235/236^ during postabsorptive conditions (*t* = 0 min), and during postprandial conditions (*t* = 120 and 360 min) after beverage intake during recovery from a single bout of concurrent exercise in young men. CASEIN, 45 g carbohydrate co-ingested with 20 g micellar casein protein; CHO, 45 g carbohydrate with 0 g protein; MILK, 45 g carbohydrate co-ingested with 20 g milk protein; mTOR, mammalian target of rapamycin; p, phosphorylated; p70S6k, ribosomal protein S6 kinase; rpS6, ribosomal protein S6; WHEY, 45 g carbohydrate co-ingested with 20 g whey protein; 4E-BP1, eukaryotic initiation factor 4E binding protein.

The phosphorylation status of mTOR^Ser2448^ (**Figure 7A**) was not different between treatments (*P* = 0.97), but was increased during the postprandial period after concurrent exercise at *t* = 120 min, but not at *t* = 360 min (*P* < 0.05). The phosphorylation status of ribosomal protein S6 kinase (p70S6k)^Thr389^ (Figure 7B) was not different between treatments (*P* = 0.69), and was not increased during the postprandial period after concurrent exercise at 120 or 360 min (*P* = 0.74). Eukaryotic initiation factor 4E binding protein 1 (4E-BP1)^Thr37/46^ phosphorylation (Figure 7C) was increased during the postprandial period after concurrent exercise at *t* = 120 min, and more so at *t* = 360 min when compared with *t* = 0 min (*P* < 0.001), with no differences among treatments (*P* = 0.95). The phosphorylation of ribosomal protein S6 (rpS6)^Ser235/236^ (Figure 7D) was increased at both *t* = 120 min and *t* = 360 min during the postprandial period after concurrent exercise when compared with *t* = 0 min (*P*= <0.01), with no difference between treatments (*P* = 0.55). Representative Western Blot images are shown in **Figure 8**.

## Discussion

In the present study, we report MyoPS and MitoPS rates after co-ingestion of MILK, WHEY, and CASEIN with 45 g carbohydrate compared with carbohydrate only (CHO) during recovery from a single bout of concurrent exercise. We report no differences in MyoPS or MitoPS rates between treatments during recovery from concurrent resistance- and endurance-type exercise, despite temporal differences in plasma amino acid availability. When MILK, WHEY, and CASEIN were collapsed into a single treatment group (PROTEIN), protein co-ingestion was shown to increase postexercise MyoPS rates, but not MitoPS rates, when compared with carbohydrate ingestion only.

Previous research has demonstrated that ingesting 20 g of a high-quality egg or whey protein maximizes postprandial MPS rates after a single bout of resistance-type exercise in young men (3, 32). However, there may be substantial differences in the capacity of different protein sources to stimulate MPS rates during recovery from resistance-type exercise (10–13, 33). Whey protein is rapidly digested and absorbed after ingestion (16, 19) and has a relatively high leucine content (∼10–12%) compared with other proteins (23). Alternatively, micellar casein has a lower leucine content (∼8%) and ingestion results in a more moderate but protracted hyperaminoacidemia (16). Milk protein, as a blend of whey and micellar casein, shows characteristics of both protein fractions (13). The rapid postprandial rise in circulating amino acids, and leucine in particular, are likely responsible for the greater postexercise MPS rates observed after whey compared with casein ingestion (10, 11). Consistent with previous observations (11, 34, 35), we found that whey protein ingestion resulted in a more rapid rise in plasma leucine concentrations (Figure 3A), with peak plasma concentrations of 322 ± 10 µmol/L (152% ± 10% increase from *t* = 0 min). In contrast, milk protein and micellar casein ingestion resulted in more moderate postprandial leucinemia (Figure 3A), with peak plasma leucine concentrations of 242 ± 8 and 245 ± 6 µmol/L (91% ± 6% and 96% ± 5% increase from *t* = 0 min), respectively. In agreement, more prolonged elevation of circulating phenylalanine and tyrosine concentrations were observed after milk protein and micellar casein ingestion compared with whey protein (Figure 3C and D). Despite substantial (transient) differences in postprandial plasma aminoacidemia (i.e., leucine, phenylalanine, and tyrosine), no differences in postprandial MyoPS or MitoPS rates were observed between CHO, MILK, WHEY, and CASEIN during recovery from a single bout of concurrent exercise (Figure 5A and 5B; Figure 6A and 6B).

This is the first study to compare postexercise MyoPS and MitoPS rates after co-ingestion of milk protein, whey, or micellar casein with carbohydrate. The lack of differences in MyoPS rates between treatments during early postexercise recovery (Figure 5A), despite markedly divergent plasma leucine concentrations (Figure 3A and 3B), is consistent with previous observations showing no differences in MyoPS rates during early postexercise recovery after ingestion of protein beverages providing 0.75, 3.0, or 5.0 g leucine (36). Also during the late recovery phase, no differences in MyoPS rates were observed between treatments. However, MyoPS rates were elevated by ∼39%, ∼27%, and ∼41% in MILK, WHEY, and CASEIN when compared with CHO during the late recovery phase (Figure 5A), which appears consistent with the notion that dietary protein-derived amino acids serve to sustain exercise-induced increases in MPS rates (37). Because there were no significant differences between treatment groups, we collapsed the data from MILK, WHEY, and CASEIN into a single treatment group (PROTEIN; *n* = 36) to assess the effect of protein-carbohydrate co-ingestion on postexercise MyoPS compared with CHO. MyoPS rates were ∼16% higher after PROTEIN compared with CHO when assessed over the aggregate 6 h of recovery from concurrent exercise (*P* = 0.04; Figure 5D). These findings seem to corroborate those of Camera et al. (27), who reported that protein ingestion (25 g whey protein) increased postprandial MyoPS rates after a single bout of concurrent exercise when compared with ingestion of a nonenergy-containing control beverage. In short, protein co-ingestion with carbohydrate results in greater postexercise MyoPS rates compared with carbohydrate ingestion only and, as such, may represent an effective nutritional strategy to facilitate skeletal muscle reconditioning after concurrent exercise.

The absence of any differences between MILK and WHEY in the current study appears consistent with the findings of Mitchell et al. (38), who reported no differences in postprandial MPS rates under nonexercised conditions after the ingestion of 20 g protein from milk or whey in middle-aged men. Similarly, the absence of any differences between WHEY and CASEIN are consistent with the findings of Tipton et al. (6) and Reitelseder et al. (14), who found no differences in the capacity of whey and casein to support a positive amino acid balance (6) or increase MPS rates (14) during recovery from resistance-type exercise. The latter was evident despite marked differences in postprandial plasma amino acid and leucine availability. In contrast, Tang et al. (11) reported greater postexercise MPS rates after the ingestion of whey compared with micellar casein. The apparent discrepancies between studies are difficult to interpret, but may relate to the processing of the applied whey or casein protein, the amount of muscle recruited during the exercise session, and/or the duration over which postprandial MPS rates were assessed during postexercise recovery. Nonetheless, the absence of differences in postexercise MyoPS rates among treatments, but benefit of protein-carbohydrate co-ingestion to increase postexercise MyoPS when compared to the ingestion of carbohydrate only, implies that milk protein, whey, and micellar casein may all be appropriate as protein sources for a carbohydrate containing beverage designed to support MyoPS rates during recovery from concurrent exercise.

Although protein ingestion stimulates increased MyoPS rates after resistance- (7, 32, 35) and endurance-type exercise (9, 39), the effect of protein ingestion on postprandial MitoPS rates in human muscle is less clear, but may also depend on the dose and/or source of ingested protein. For example, Walrand et al. (40) recently reported that ingestion of soluble milk protein but not casein protein increased postprandial MitoPS rates in healthy elderly men, demonstrating that the type of ingested protein is important when considering the impact of protein intake on postprandial MitoPS rates. However, we found no difference in MitoPS rates in response to MILK, WHEY, and CASEIN during postexercise recovery (Figure 6A and 6B). When we collapsed the data from MILK, WHEY, and CASEIN into a single treatment group (PROTEIN; *n* = 36), MitoPS rates were a nonsignificant ∼13% higher in response to PROTEIN when compared with CHO when assessed over the aggregate 6-h recovery period after concurrent exercise (*P* = 0.20; Figure 6D). The lack of difference in MitoPS rates between PROTEIN and CHO in the current study is in line with previous studies that found no difference in postprandial MitoPS rates after protein ingestion before (29) or after exercise (9, 27, 28) when compared with MitoPS rates measured in response to exercise or protein ingestion only. Recent research has demonstrated increased postprandial MitoPS rates under resting conditions after the ingestion of 36 g (41), but not 18 g (28) of protein, hence future studies should evaluate whether MitoPS rates are increased in response to greater doses (e.g., 30 g) of ingested protein after exercise.

In the present study, we compared postprandial MyoPS and MitoPS rates after ingestion of different types of protein (20 g) when co-ingested with ample carbohydrate (45 g). Comparing these protein sources within the context of carbohydrate co-ingestion was chosen for pragmatic reasons as the Academy of Nutrition and Dietetics, Dietitians of Canada, and the American College of Sports Medicine currently recommend that nutritional strategies to promote postexercise recovery should include both carbohydrate and protein intake (42). Besides ingesting protein to support postexercise adaptation, repair, and remodeling, a rapid restoration of depleted muscle glycogen is an important target to facilitate recovery from endurance-type exercise (43). To date, no studies have compared the capacity of different sources of dietary protein to increase postexercise MPS rates when co-ingested with ample carbohydrate. We have recently shown that carbohydrate co-ingestion with protein can attenuate dietary protein digestion and absorption kinetics, thereby blunting the postprandial rise in aminoacidemia when compared with protein ingestion only (44). Given the marginal increase in postexercise MyoPS, and lack of stimulation in postexercise MitoPS rates after ingesting 20 g protein, we speculate that higher protein doses (e.g., 30 g) may be needed to induce a more robust stimulation of MyoPS and MitoPS rates when protein is co-ingested with carbohydrate during recovery from concurrent exercise.

All previous studies to date examining the impact of protein source on postprandial MPS rates after exercise in humans have been conducted after single-mode resistance-type exercise (6, 11, 13, 14, 33). The present study is the first to examine the impact of the source of ingested protein on postprandial MyoPS and MitoPS rates after concurrent exercise. Here, we observed substantial variation in the individual response to the combined ingestion of protein and carbohydrate on postprandial MyoPS (Figure 5; panels A–D) and MitoPS (Figure 6; panels A–D) rates during recovery from concurrent exercise. In agreement, Camera et al. (27) reported a large, unexpected variability in MyoPS and MitoPS rates during recovery from concurrent exercise. Combining resistance- and endurance-type exercise in concurrent exercise training has been reported to attenuate gains in muscle mass, strength, and power when compared with exercise training composed of single-mode resistance-type exercise (45). This phenomenon observed during concurrent exercise training has been coined the “interference effect” (46). As previously suggested by Camera et al., successive bouts of resistance- and endurance-type exercise may increase the complexity of the genotype-exercise interaction in promoting the adaptive response of skeletal muscle, with individual responses to nutritional interventions adding a further level of complexity (27).

Consistent with our findings on postprandial MyoPS and MitoPS rates after exercise, the phosphorylation status of the intracellular signaling proteins we measured did not differ between treatments (Figure 7; panels A–D). Although it is unequivocal that amino acids enhance signaling through mTORC1 (5), our measurements of the phosphorylation status mTOR^Ser2448^, p70S6k^Thr389^, 4E-BP1^Thr37/46^, and rpS6^Ser235/236^ were all made under postexercise conditions, and all treatments provided ample amounts of carbohydrate. Both single-mode resistance- (47) and endurance-type exercise (48, 49) serve as potent contractile stimuli to increase mTORC1 and its downstream targets in human muscle. Furthermore, insulin (e.g., via carbohydrate intake) can activate mTORC1 via Akt (50). Therefore, performance of an acute bout of concurrent exercise coupled with carbohydrate intake and increased insulin availability may have already increased the phosphorylation status of these proteins, thereby masking amino acid–induced changes in mTORC1 and its downstream targets. Alternatively, concurrent exercise may have already increased amino acid availability to the muscle by increasing endogenous amino acid release, and/or by stimulating skeletal muscle blood flow. These factors may have made the contribution of the postprandial release of exogenous amino acids less relevant to the changes in mTORC1 and its downstream targets. Another possibility is that the endurance-type exercise bout in the current study interfered with mTORC1 related signaling responses to the prior bout of resistance-type exercise, an effect demonstrated in rodents (51), but not currently supported in humans (52). Protein ingestion may not have been able to overcome such a potential inhibitory effect of endurance exercise on mTORC1-related signaling responses. Lastly, because the first biopsy obtained after treatment administration did not occur until *t* = 120 min into the postprandial period, we may simply have missed any divergent signaling responses between treatments that may have occurred before this time point.

In conclusion, MyoPS and MitoPS rates do not differ after co-ingestion of milk protein, whey protein, or micellar casein protein with carbohydrate during recovery from a single bout of concurrent resistance- and endurance-type exercise in recreationally active young men. Protein-carbohydrate co-ingestion results in greater postprandial MyoPS rates, but not MitoPS rates, when compared with carbohydrate ingestion only, and as such, may represent an effective nutritional strategy to support skeletal muscle reconditioning after concurrent exercise.

## Supplementary Material

nxy244_Supplemental_Files

## Notes

Supported in part by PepsiCo/Gatorade Sports Science Institute.

Supplemental Tables 1 and 2 and Supplemental Methods are available from the “Supplementary data” link in the online posting of the article and from the same link in the online table of contents at https://academic.oup.com/jn/.

Abbreviations used: CASEIN, 45 g carbohydrate co-ingested with 20 g micellar casein protein; CHO, 45 g carbohydrate with 0 g protein; FSR, fractional synthetic rate; IAA, indispensable amino acids; MILK, 45 g carbohydrate co-ingested with 20 g milk protein; MitoPS, mitochondrial protein synthesis; MPS, muscle protein synthesis; mTOR, mammalian target of rapamycin; mTORC1, mammalian target of rapamycin complex 1; MyoPS, myofibrillar protein synthesis; PROTEIN, 45 g carbohydrate co-ingested with 20 g protein, data collapsed across MILK, WHEY, and CASEIN; WHEY, 45 g carbohydrate co-ingested with 20 g whey protein; 1-RM, 1 repetition maximum.
