# Supplementary material for: Myofibrillar and Mitochondrial Protein Synthesis Rates Do Not Differ in Young Men Following the Ingestion of Carbohydrate with Milk Protein, Whey, or Micellar Casein after Concurrent Resistance- and Endurance-Type Exercise
Source: J Nutr. 2019 Jan 29;149(2):198–209. doi: 10.1093/jn/nxy244 (PMC6561606; doi:10.1093/jn/nxy244)
Supplement: nxy244_Supplemental_Files [file nxy244_supplemental_files.zip › Churchward-Venne et al. 2018A - Supplemental Table 1.pdf]

Supplemental Table 1. Characteristics of young recreationally active men who ingested nutritional treatments consisting of carbohydrate only, or carbohydrate co-ingested with milk, whey, or micellar casein protein following a single bout of concurrent exercise[1].

	Nutritional treatment group				
	CHO	MILK	WHEY	CASEIN	*P* value
Age (y)	23±1	24±1	23±1	24±1	0.85
Height (m)	1.80±0.01	1.80±0.02	1.80±0.01	1.79±0.02	0.98
Weight (kg)	74.5±2.0	73.6±2.3	76.0±2.0	72.8±2.5	0.76
BMI (kg/m^2)	22.9±0.4	22.6±0.6	23.4±0.6	22.6±0.6	0.69
Fat + bone-free mass (kg)	57.4±1.4	57.3±1.9	58.6±1.5	57.0±1.9	0.92
Fat mass (kg)	15.1±1.2	14.4±0.8	15.3±0.9	14.1±1.1	0.81
% Fat	19.8±1.2	19.3±0.9	19.8±0.9	18.9±1.1	0.91
Systolic BP (mmHg)	129±2	128±3	123±3	127±3	0.56
Diastolic BP (mmHg)	70±3	70±3	64±4	66±2	0.53
Leg Press 1-RM (kg)	253±13	260±17	271±13	259±14	0.85
Leg Extension 1-RM (kg)	118±6	122±6	121±5	120±6	0.96
Maximal workload (W)	261±10	262±15	266±13	278±10	0.73

[1]Values are mean ± SEM. n = 12. Data were analyzed using a 1-factor ANOVA. CHO, 45 g carbohydrate with 0 g protein; MILK, 45 g carbohydrate co-ingested with 20 g milk protein; WHEY, 45 g carbohydrate co-ingested with 20 g whey protein; CASEIN, 45 g carbohydrate co-ingested with 20 g micellar casein protein.
