# Supplementary material for: Myofibrillar and Mitochondrial Protein Synthesis Rates Do Not Differ in Young Men Following the Ingestion of Carbohydrate with Milk Protein, Whey, or Micellar Casein after Concurrent Resistance- and Endurance-Type Exercise
Source: J Nutr. 2019 Jan 29;149(2):198–209. doi: 10.1093/jn/nxy244 (PMC6561606; doi:10.1093/jn/nxy244)
Supplement: nxy244_Supplemental_Files [file nxy244_supplemental_files.zip › Churchward-Venne et al. 2018A - Supplemental Table 2.pdf]

Supplemental Table 2. Amino acid (L-form), protein, and carbohydrate contents of nutritional treatments consisting of carbohydrate only, or carbohydrate co-ingested with milk, whey, or micellar casein protein following a single bout of concurrent exercise in young recreationally active men [1].

	Nutritional treatment group			
	CHO	MILK	WHEY	CASEIN
**Amino acid content**				
Alanine, g	-	0.54	1.02	0.62
Arginine, g	-	0.71	0.62	0.72
Asparagine, g	-	1.32	2.36	1.50
Cysteine, g	-	0.13	0.56	0.12
Glutamine, g	-	3.55	3.66	4.24
Glycine, g	-	0.31	0.38	0.36
Histidine, g	-	0.47	0.44	0.56
Isoleucine, g	-	0.88	1.14	1.00
Leucine, g	-	1.65	2.58	1.96
Lysine, g	-	1.38	2.14	1.62
Methionine, g	-	0.48	0.48	0.58
Phenylalanine, g	-	0.81	0.78	1.04
Proline, g	-	1.78	1.02	2.06
Serine, g	-	0.96	0.94	1.14
Threonine, g	-	0.76	1.08	0.88
Tryptophan, g	-	0.22	0.42	0.26
Tyrosine, g	-	0.87	0.74	0.54
Valine, g	-	1.16	1.06	1.26
**Totals**				
ΣNEAA, g	-	10.18	11.30	11.30
ΣEAA, g	-	7.81	10.12	9.16
ΣAA, g	-	17.99	21.42	20.46
Protein, g	-	20.00	20.00	20.00
Carbohydrate, g	45.00	45.00	45.00	45.00

[1]Note: Total protein was calculated as Nitrogen content × 6.38. CHO, 45 g carbohydrate with 0 g protein; MILK, 45 g carbohydrate co-ingested with 20 g milk protein; WHEY, 45 g carbohydrate co-ingested with 20 g whey protein; CASEIN, 45 g carbohydrate co-ingested with 20 g micellar casein protein.
