# Supplementary material for: Myofibrillar and Mitochondrial Protein Synthesis Rates Do Not Differ in Young Men Following the Ingestion of Carbohydrate with Milk Protein, Whey, or Micellar Casein after Concurrent Resistance- and Endurance-Type Exercise
Source: J Nutr. 2019 Jan 29;149(2):198–209. doi: 10.1093/jn/nxy244 (PMC6561606; doi:10.1093/jn/nxy244)
Supplement: nxy244_Supplemental_Files [file nxy244_supplemental_files.zip › Churchward-Venne et al. 2018A -Supplemental Methods.pdf]

Supplemental data

Supplemental methods

Plasma analysis

Plasma glucose and insulin concentrations were analyzed by *Dr. Stein und Kollegen Laboratories* (Mönchengladbach, Germany) using commercially available kits (GLUC3, Roche, Ref: 05168791 190, and Immunologic, Roche, Ref: 12017547 122, respectively). Plasma leucine, phenylalanine, and tyrosine concentrations as well as plasma L-[ring-$^{13}C_6$]-phenylalanine, L-[ring-$^{13}C_6$]-tyrosine, and L-[ring-3,5-$^{2}H_2$]-tyrosine enrichments were determined by gas chromatography–mass spectrometry (GC-MS; Agilent 7890A GC/5975C MSD; Agilent Technologies). Specifically, internal standards of [U-$^{13}C_6$]-leucine, [U-$^{13}C_9$ ^{15}N]-phenylalanine, and [U-$^{13}C_9$ ^{15}N]-tyrosine were added to the plasma samples. The plasma was deproteinized on ice with dry 5-sulfosalicylic acid. Free amino acids were purified using cation exchange AG 50W-X8 resin (mesh size: 100-200, ionic form: hydrogen (Bio-Rad Laboratories, Hercules, CA)) columns. The free amino acids were converted to their tert-butyl dimethylsilyl (TBDMS) derivative before analysis by GC-MS. The amino acid concentrations were determined using electron impact ionization by monitoring ions at mass/charge (m/z) 302 and 308 for unlabeled and [U- $^{13}C_6$]-leucine respectively, 336 and 346 for unlabeled and [U- $^{13}C_9$ ^{15}N]-phenylalanine respectively, and 466 and 476 for unlabeled and [U-$^{13}C_9$ ^{15}N]-tyrosine respectively. For plasma L-[ring-$^{13}C_6$]-phenylalanine enrichment measurements, plasma phenylalanine was derivatized to the TBDMS derivative, and the ^{13}C enrichments were determined by gas chromatography-mass spectrometry (GC-MS; Agilent 7890A GC/5975C MSD; Agilent Technologies) using selected ion monitoring of masses 336 and 342 for unlabelled and labelled [U- $^{13}C_6$]-phenylalanine, respectively. Standard regression curves were applied from a series of known standard enrichment values against the measured values to assess the linearity of the mass spectrometer and to account for any isotope fractionation which may have occurred during the analysis. Phenylalanine and tyrosine enrichments were corrected for the presence of both the ^{13}C and ^{2}H isotopes.

Muscle analysis

The myofibrillar enriched protein pellet was washed twice with the homogenization buffer and centrifuged at 700*g* for 5 min at 4°C. The supernatant was discarded. The myofibrillar enriched proteins were solubilised by adding 1.5 mL of 0.3 M NaOH and heating to 37°C for 30 min with vortex mixing every 10 min. Samples were centrifuged at 700*g* for 5 min at 4°C and the supernatant containing the myofibrillar-enriched fraction was collected and the collagen pellets were discarded. Myofibrillar proteins were precipitated by the addition of 1 mL of 1 M PCA and centrifuged at 700*g* for 10 min at 4°C. The myofibrillar enriched protein fraction was washed twice with 70% ethanol. The amino acids were liberated from the myofibrillar enriched protein fraction by adding 1.5 mL of 6 M HCl and heating to 110°C for 24 h. The mitochondrial enriched protein pellet was washed twice with 500 µl of an ice-cold homogenizing buffer (1 m sucrose, 1 M Tris/HCl, 1 M KCl, 1 M EGTA/Tris) and centrifuged at 12,000 *g* for 5 min at 4°C. The supernatant was discarded and the pellet was washed with 95% ethanol and centrifuged at 12,000 *g* for 5 min. The supernatant was discarded and the pellet was lyophilized. Amino acids were liberated from the mitochondrial enriched protein fraction by adding 1.5 mL of 6 M HCl and heating to 110°C for 24 h. The hydrolysed myofibrillar and mitochondrial protein fractions were dried under a nitrogen stream while heated to 120° C, then dissolved in a 50% acetic acid solution.

Supplemental data

The amino acids from the myofibrillar protein fraction (but not the mitochondrial protein fraction) were passed over a Dowex exchange resin (AG 50W-X8, 100-200 mesh hydrogen form; Bio-Rad, Hercules, CA, USA) using 2 M NH_4OH. The amino acids from the mitochondrial and myofibrillar protein fractions were derivatized to their N(O,S)-ethoxycarbonyl-ethylesters. The ratio of $^{13}C/^{12}C$ of muscle protein-bound phenylalanine was determined using gas chromatography-combustion-isotope ratio mass spectrometry (GC-IRMS; Delta V, Thermo Scientific, Bremen, Germany) by monitoring ion masses 44, 45 and 46. The $^{13}C/^{12}C$ abundance ratio was expressed as $\delta^{13}C$ values calibrated against the international standard of Vienna Pee Dee Belemnite (VPDB). Standard regression curves were applied to assess linearity of the mass spectrometer and to control for the loss of tracer.

For Western blot analysis, the supernatant was heated for 5 min at 100°C and immediately placed on ice. Immediately before analyses, the muscle extraction sample was warmed to 50°C and centrifuged for 1 min at 1,000g [room temperature (RT)]. Total amount of sample loaded on the gel was based on weight (35 µg/lane). The protein concentration of each sample was determined via the Pierce BCA protein assay kit (prod nr. 23225, Thermo scientific, Rockford, USA). With the exception of mammalian target of rapamycin (mTOR), protein samples were run on a Criterion Precast TGX 4–20% gel (Bio-Rad order no. 567-1094) for 10 min at 50 V (constant voltage) and ±90 min at 150 V (constant voltage) and transferred onto a Trans-blot Turbo 0.2-µm nitrocellulose membrane (Bio-Rad order no. 170-4159) for 7 min at 2.5 A and 25 V. mTOR proteins were run and blotted under the same conditions but on a Criterion Precast XT 3–8% Tris-acetate gel (Bio-Rad order no. 345-0130). Specific proteins were detected by overnight incubation at 4°C on a shaker with specific antibodies in 50% PBS Odyssey blocking buffer (part no. 927-40000; LI-COR Biosciences, Lincoln, NE) after blocking for 60 min at RT in 50% PBS Odyssey blocking buffer. Specific proteins were detected with the following antibodies: anti-mTOR (289 kDa, dilution 1:1,000, no. 2972; Cell Signaling Technology, Danvers, MA) and anti-phospho-mTOR (^{Ser2448}, 289 kDa, dilution 1:1,000, no. 2971; Cell Signaling Technology), anti-p70S6k (70 kDa, dilution 1:1,000, no. 9202; Cell Signaling Technology) and anti-phospho p70S6k (^{Thr389}, 70 kDa, dilution 1:1,000, no. 9206; Cell Signaling Technology), anti-rpS6 (32 kDa, dilution 1:1,000, no. 2217; Cell Signaling Technology) and anti-phospho-rpS6 ($^{Ser235/236}$, 32 kDa, dilution 1:1,000, no. 4856; Cell Signaling Technology), and anti 4E-BP1 (15/20 kD, dilution 1:1,000, no. 9452; Cell Signaling Technology) and anti phospho 4E-BP1 ($^{Thr37/46}$, 15-20 kD, dilution 1:1,000, no. 9459; Cell Signaling Technology). Following incubation, membranes were washed three times for 10 min in 0.1% PBS-Tween 20 and once for 10 min in PBS. Next, samples were incubated on a shaker (1 h at RT) and complementary secondary antibodies were applied (IRDye 800 donkey anti-rabbit (cat. No. 926-32213, dilution 1:10,000; LI-COR Biotechnology, Lincoln, NE) and IRDye 800CW donkey anti-mouse (cat. no. 626-32212, dilution 1:10,000; LI-COR Biotechnology)) dissolved in 50% PBS Odyssey blocking buffer. After a final wash step (3 × 10 min) in 0.1% Tween 20-PBS and once for 10 min in PBS, protein quantification was performed by scanning on an Odyssey Infrared Imaging System (LI-COR Biotechnology, Lincoln, NE), with local background subtraction and intensity of the bands expressed as fold-change from *t*=0 min. Phosphorylation status as a proxy of activation of the signaling proteins was expressed relative to the total amount of each protein.
